# Views of Israeli healthcare professionals regarding communication of genetic variants of uncertain significance to patients

**DOI:** 10.1002/jgc4.1560

**Published:** 2022-02-04

**Authors:** Alma Levin Fridman, Aviad Raz, Stefan Timmermans, Shiri Shkedi‐Rafid

**Affiliations:** ^1^ Department of Sociology and Anthropology Ben‐Gurion University of the Negev Be'er Sheva Israel; ^2^ Department of Sociology UCLA Los Angeles California USA; ^3^ Department of Genetics Hadassah Medical Organization and Faculty of Medicine Hebrew University of Jerusalem Jerusalem Israel

**Keywords:** communication, genomic designation, uncertainty, variant classification, variant of uncertain significance

## Abstract

While genomic medicine is becoming an important part of patient care with an ever‐increasing diagnostic yield, communicating variants of uncertain clinical significance (VUSs) remains a major challenge. We draw on qualitative analysis of semi‐structured interviews conducted in 2020 with 20 Israeli healthcare professionals and stakeholders involved in communicating the results of genome‐wide sequencing to patients. Respondents described four main strategies of communicating VUSs to patients: preparing the patient pre‐test for uncertainty; adapting the level of detail to the patient's needs; upgrading versus downgrading the VUS; and following up on the possible reclassification of VUSs. These strategies were expressed differently by physicians and genetic counselors, varying according to their specialty and perception of the patient's situation. We discuss the strategic management and communication of uncertain genomic test results with patients in the context of meeting patients' expectations and working toward genetic causality through genomic narration and designation.

## 1 INTRODUCTION

New technologies of DNA sequencing produce increasingly more high‐resolution genetic data. Next‐generation sequencing technologies provide in a single test more data on more genes or regions in the genome than the earlier sequencing technologies. Consequently, variants of uncertain significance are found more often, leading to a continuous process of re‐interpreting and reclassifying genetic variants as either benign or pathogenic. Recent research suggests that about 8% of the variants initially classified as variants of uncertain clinical significance (VUSs) were later reclassified: either downgraded to less severe classification (about 91% of variants) or upgraded to more severe classifications (about 9% of variants, see Mersch et al., 2018). Reclassification is based on new information regarding variants' pathogenicity, such as population frequency, functional data, segregation analysis, and phenotype analysis. Periodical reinterpretation of VUSs is therefore inevitable yet involves collaboration among multiple stakeholders: public and private genetics laboratories, clinicians, genetic health professionals, and medical geneticists (Carrieri et al., 2017). It is challenging for healthcare professionals (HCPs) to remain abreast of VUS reclassification.

Communicating and interpreting VUSs is trying for both patients and HCPs (Clift et al., 2020; Stivers & Timmermans, 2016; Timmermans et al., 2017; Zhong et al., 2021) and poses different challenges across medical settings and indications. In the context of prenatal genetic diagnosis, some prospective parents have reported feeling anxious after receiving a VUS result and considered the uncertain and unquantifiable risks ‘toxic knowledge’ that caused lingering worries about their child's development (Bernhardt et al., 2013). Another study found that following the initial shock, parents had no enduring concerns about VUSs (van der Steen et al., 2016). Cancer patients who were more worried about the course of their disease disregarded VUSs (Clift et al., 2020). Finally, patients with undiagnosed symptoms reacted positively to VUSs which were considered as a potential hint that may lead to future diagnosis (Kiedrowski et al., 2016; Skinner et al., 2017). HCPs’ and patients’ responses to uncertainty, therefore, are not simply a function of the abstract uncertainty itself; they are subjectively shaped by the contingent meanings given to that uncertainty within the specific contexts of a diagnostic odyssey (Werner‐Lin et al., 2016, 2018). Genetic counselors (GCs) are often the main professional authority for patients to make sense of genetic uncertainty, and as a result, GCs often feel a need to manage uncertainty in a way that allows them to facilitate collaborative decision‐making regarding treatment, follow‐up, prevention, and testing other family members (Rauscher, 2017).

Communicating with patients about VUSs is paradoxical: Each VUS, by nature of its liminal, temporal qualities, invites communication about what is still uncommunicable due to inherent gaps of data and understanding. Therefore, the VUS requires narration (Timmermans et al., 2017) and designation (Navon, 2019). These terms, which do not have a strict definition, refer to communication efforts that attempt to create meaning from uncertain results. This includes an effort to fit the VUS into relevant genetic models, for example of expressed disease characteristics in biological family members, even though the associated phenotype is not distinct enough for diagnosis. The VUS may lead HCPs and their patients down any number of paths, including some dead ends. Communication and uncertainty are known to go hand in hand in health care (Mishel, 1990). Yet, in the case of VUSs, uncertainty is at the core of communication itself. HCPs must negotiate the expectations of patients, including negotiating how communication of uncertainty affects patients’ perceptions of the HCPs' credibility (Zhong et al., 2020). Furthermore, studying communication strategies of VUS disclosure has important implications for how genetic counselors can help facilitate meaning‐making for patients (Scherr et al., 2020). This study seeks to examine HCPs' communication methods and challenges regarding the disclosure of VUSs in clinical care, a topic which we still know relatively little of and, as a result of its recent introduction into practice, does not have clear professional guidelines. In a context of significant clinical uncertainty, for both experts and patients receiving VUS from panel testing, identifying communication processes is an essential step toward developing guidelines and supporting interventions for decisions that will become increasingly common as panel and exome testing spreads.

Uncertainty is usually constructed as negative in medical training (Atkinson, 1984; Han et al., 2017; Lupton, 2013; Newson et al., 2016). Nevertheless, team discussions of uncertainty, including that related to VUS, can be used productively as a catalyst for social action (Timmermans et al. (2017). VUS communication can keep patients under medical purview in an attempt to establish genetic causality, especially when facing patients who are searching for a cause of their symptoms, specifically tumor profiling oncology, cardiomyopathy, and undiagnosed‐disease genetic testing patients (Clift et al., 2020).

To provide much needed empirical evidence about communication of VUS in the fields of cancer and cardio genetics (where panel and exome testing is being integrated into care thus entailing routine discovery of VUSs), this paper draws on interviews with Israeli healthcare professionals involved in genomic medicine. These were chosen as they are involved in precision genomic medicine either as clinicians referring to testing, interpreting genomic results, and following up patients. Identifying the strategies that HCPs and particularly GCs use to communicate uncertainty should help advance research on communication and counseling in precision genomic medicine.

## 2 METHODS

### 2.1 Study design

Semi‐structured interviews were conducted as part of a broader study to investigate ethical and social issues of negotiating genome‐wide sequencing (GWS) in clinical care.

### 2.2 Recruitment and sample

Following IRB approval, we contacted relevant (mostly oncogenetic) physicians and genetic counselors from 6 different medical centers across Israel. Participants were identified and invited to participate by the clinical author of this paper (SSR) based on her professional experience and networks. The first author (a female MA student in medical sociology with training in qualitative methodology and an interest in the research topic) conducted all the interviews. Interviews lasted between 30 and 60 min, and given the COVID‐19 pandemic circumstances, were mainly conducted via telephone or online. No one else was present in the interviews besides the participants and the researcher. All those approached agreed to take part in the research. None of the researchers or the participants expressed a need to carry out repeat interviews. Where needed, the interviewer made field notes after the interview, elaborating for example on the interview data, setting, and circumstances.

### 2.3 Procedures

Based on relevant literature and the clinical experience of the fourth co‐author, the research team (composed of two male researchers and two female researchers) prepared the guide for the semi‐structured interviews, including questions addressing the following topics: (a) how medical geneticists, genetic counselors, and clinicians cope with the challenge of uncertainty in genetic testing results? and (b) What strategies do HCPs use to communicate uncertainty regarding VUSs results to patients? Specific questions addressed experiences and challenges concerning communicating VUSs with patients (see Table 1). Additional questions concerning raw data and incidental findings which were part of the guide are not discussed here.

**TABLE 1:** Semi‐structured interview guide

Welcome and Introduction: Tell me about your professional specialty. How long have you had that specialty? How do you use genomic tests in your daily work?
1. Are uncertain genetic findings a salient part of your job?
2. How do you decide whether to deliver uncertain test results to patients?
3. What, if any, recommendations do you provide to patients when telling them about VUSs?
4. When do you recommend involving other family members regarding inquiries about VUS?
5. Do you keep yourself up to date regarding a VUS, or are you satisfied with the information provided in the lab report?
6. How do patients react to VUSs?
7. How do you present the VUS to patients ‐ in an optimistic, pessimistic, or neutral manner?
8. How do you prepare patients for the possibility of VUS?
9. Can you describe examples in which you talked with patients about a VUS as likely pathogenic?
10. Do you talk with patients about following up on VUS reclassification as the responsibility of the patient, the lab, the clinician, or the genetic counselor?
11. Do you think patients should be given the option to opt‐out of receiving information about VUS?

### 2.4 Data analysis

The anonymized transcripts were analyzed thematically to uncover discursive themes and categories of themes recurring within and across groups of respondents, for example, groups of different HCPs (Charmaz & Belgrave, 2012). We followed an exploratory, qualitative methodology, which is aimed at advancing existing theory using unstructured, open‐ended data, and is thus particularly appropriate for the current investigation. Following qualitative data analysis methods, our approach was to develop themes by constantly making comparisons and noting relationships among initially identified codes, inductively specifying and refining these codes, and then putting them in thematic categories or families. This iterative, reflective practice enables to build new theories or to extend existing theory on issues that have been largely understudied (Charmaz & Belgrave, 2012).

Following a review of the relevant literature, preliminary codes included communication strategies, consent, and responsibility to recontact ('following up'). The research team discussed the initial interviews and agreed that they pertained to these codes, which were then analyzed further inductively. The texts pertaining to each of these codes were extracted, and then in the next rounds of the analysis and discussions, more specific themes within the text pertaining to these preliminary codes were identified, as well as new themes that emerged from the analysis. For example, the preliminary, general code of 'communication strategies' was refined into several specific strategies, including preparing the patient for uncertainty, adapting the level of detail, down‐ or upgrading the VUS, and following up on the possible reclassification of VUSs. These more specific strategies of communicating VUSs were thereafter treated as themes, each with its relevant set of codes. Through such inductive analysis, 'communication strategies' which were treated to begin with as a preliminary code were replaced by more refined coding and came to be regarded as a broad thematic category. The research team did the coding together on the first few interview transcripts, discussing the relevance of the themes and agreeing on needed modifications and reclassifications. The first author completed the coding of the remaining transcripts. In this manner, we collected data and analyzed them simultaneously, starting with the initial interviews and their analysis and following with an additional round of interviews and their analysis. Following the analysis of the first round of the interviews, we made some mild changes to some of the questions, adjusting the phrasing for clarity and simplicity. We did not use a specific software for qualitative analysis. Participants were able to receive their interview transcripts for comment and/or correction, yet none of the participants requested this.

We discussed new findings as they appeared and their relationships to the codes in team meetings, where agreements were reached to prevent the potential bias of a single rater. The iterations stopped after the first ten interviews, when the authors agreed on all the themes and no new themes were identified, suggesting that theoretical saturation of the sample was achieved (Corbin & Strauss, 2008). Each of the themes is described below and illustrated with quotes from respondents, who are given pseudonyms. These quotes were translated by the authors from Hebrew to English. Quotes were selected because they were noted by at least two of the authors as examples that best captured the identified themes. We did not conduct participant checking since in studies that are not participatory or collaborative there is little evidence that member checks improve research findings (Thomas, 2017). We focus here on views presented concerning communication pathways regarding the initial presentation of VUSs. Due to space restrictions, we cannot elaborate here on communication regarding the follow‐up of VUSs reclassification and recontact, a topic we focus on elsewhere.

## 3 RESULTS

The sample was comprised of healthcare professionals who communicate GWS results to patients: 8 genetic counselors (two of them with cardio‐genetic expertise), 5 medical geneticists (3 specialized in cardio‐genetic), 5 oncologists, and 2 legal experts with bioethical expertise concerning GWS in Israel (total *n* = 20). All participants had more than 5 years of experience in their specialty. All participants were white non‐Hispanic and worked in a public medical center (except for the 2 bioethicists) (see Table 2 for experts' demographics).

**TABLE 2:** Expert demographics (*N* = 20)^a^

Demographics	Value
Years of practice, mean (range)	11.4 (5–30)
Training	
Genetic counselors (board certified)	8 (40)
Onco‐genetics specialty	6 (30)
Cardio‐genetics specialty	2 (10)
Physicians (MD)	10 (50)
Oncology	5 (25)
Internal/Pediatric with genetics sub‐specialization	5 (25)
Cardiology	1 (5)
Bioethicists	2 (10)
Genetics training	
Yes	15 (75)
No^b^	5 (25)
Gender	
Male	6 (30)
Female	14 (70)

^a^ Values are presented as number (%) unless otherwise indicated.

^b^ Two respondents who indicated ‘no’ were bioethicists, and the other three were oncologists who had no genetics training.

Five core themes were defined: The impact of VUSs on patient care; preparing the patient for uncertainty; adapting the level of detail; down‐ or upgrading the VUS; and following up. We mention the first theme in brief as it provides the broader context for communicating VUSs to patients and focus in more detail on the other four themes to highlight the variety of communication strategies used by HCPs.

### 3.1 The impact of VUSs on patient care

While all our respondents agreed that VUSs presented a challenge by virtue of their liminal and uncertain nature, their views of the extent of that challenge varied by their specialty and experience. A common view among the oncologists, for example, was that VUSs are not that important for improving care or to communicate to patients:

Generally speaking, for cancer patients, VUSs are one of the less interesting things to talk about [Ali, oncologist]

If it's a somatic VUS that does not predict cancer risk and I think it's meaningless, I would not go into it [with the patient] in detail […] If the VUS might have consequences for family members then I refer the patient to genetic counseling. Let the genetic counselors worry about it [Cole, oncologist]

The genetic counselors were overall more concerned (in comparison with the physicians) with what they perceived as 'their' responsibility of correctly evaluating the clinical significance of VUSs and the subsequent recommendations given to patients. However, some of them viewed this challenge as something that, with time, has become more familiar and less worrisome. As Carrol, a GC, typically described:

We have become gradually more accustomed to VUSs over the years […] In the beginning, every VUS was stressful and exciting, but in time we learned that almost all the test results have VUSs in them and the vast majority of VUSs are probably benign […] As for the approximately 10 per cent of VUSs that may become potentially reclassified as pathogenic, this only clarifies the importance of keeping in touch with the patient.

In addressing the challenge of trying to make sense of a VUS, a few GCs were critical of communicating to patients what they saw as 'overly interpretive' reclassification of VUSs. Monica, a GC specialized in cardiology, described a case in which a VUS that was related to heart diseases was found in a patient who died of a heart attack at a young age. After many consultations among the HCPs, it was decided that other asymptomatic family members should be tested for the VUS, and those found to carry it needed to undergo surveillance. Many years later, she said not one of those family members had heart problems but *'the negative consequences of the labeling were real*'. For example, the brother of the deceased patient apparently was not recruited to military service because of the VUS. The GC summed up the lesson of that case by saying: *'If you're in doubt, do not make screening recommendations to patients [based on a VUS]. That's a really important principle’*.

### 3.2 Preparing the patient for uncertainty

Most HCPs explained that they prepare their patients in advance, before the genetic test is conducted, regarding the possibility of receiving uncertain results. All the GCs, as well as most of the clinicians, said they explain this possibility when talking with the patient about the genetic test. Amy, a GC, explained this in a typical manner:

I stress before going on with the test that results come in three options, and it's very important they [patients] understand this: benign, not benign, these are the obvious options, but there is also the option of in‐between. They do not know such an option of in‐between exists and it is important to explain about it. When I give them the results I say: 'remember we spoke about results that are gray, in‐between, unknown? So, we got one of those’. Then it's much easier because they knew it was a possibility and could have prepared for it.

Furthermore, most HCPs agreed that in preparing the patients for VUSs, they usually underplayed their importance. HCPs' presentation of the option of VUSs ranged from the dismissal of the significance of VUSs to stressing the unlikelihood that VUSs would convey a concrete risk. Several HCPs said that they tell patients that a VUS for them is a good, normal result, that is, downplaying the VUS. As Rose, a GC, expressed it: ‘*I don't make a fuss because I know that if it's a VUS I will not make a fuss about it. I barely talk about it. I just tell them it's normal’*. Anika, an oncologist, commented that she prepares patients in the following way:

Usually, I tell patients before sending them to a genetic panel [test] that there are three groups of results: black, white, and gray. I also tell them that statistically speaking, we get 30 percent VUS or gray results. Some patients say 'I don’t want to live with something gray like this over me. If it's not black or white I'd rather not take the test’.

The last quote raises the question of informed consent/choice regarding opting out from receiving VUSs as results. Currently, the official consent forms used by HCPs in Israel (as in most other countries) do not offer opting out from receiving VUSs in the oncological and cardiovascular clinical setting. However, most respondents, including most GCs, agreed that they would let the patient express their wish about receiving VUSs and in most cases will respect that wish. A minority view expressed by a few clinicians was that if the patient consents to the test, it implies legally and practically that they consent to receive VUSs. Zelda, an oncologist, typically explained this position in the following words:

I do not agree to have relevant data that I cannot relay to a patient because that patient did not understand some legal clause. This is my opinion. It's the patient's right not to be tested, it's their choice if they want to know or not. But once you know something about them, even a VUS, not telling them sounds cruel to me.

### 3.3 Adapting the level of detail

All HCPs said they talk with patients about VUSs, except for two oncologists who said they refer their patients to GCs for VUSs consultations. Most HCPs said they talk about VUS without going into too much detail unless they are suspicious of that VUS. As Amy, a GC, typically explained:

I tell them it's a finding, but I do not practically give details. I try to push them away from it [the VUS] so they don't fuss about it, because really, I don’t want them to make decisions based on VUSs

Rose, a GC, was critical of talking 'too much' about a VUS:

I think that genetic counselors have an hour for a consultation so they let themselves talk away until the patient gets all confused. I think it's bad, at the end of the day the patients walk away without better understanding.

A few of the GCs said they explain about VUSs in detail. Warner, a GC, said that:

I explain all I can possibly say about the VUS […] its frequency in the population and among other patients […] I do it even if the patient doesn't really understand

Peter, a medical geneticist, explained that she decides how detailed she should be according to her assessment of the patient:

I assess the patient's ability to understand what I'm saying. This is paternalistic, but I think it's not so bad to be paternalistic in this situation. Some people can understand VUS and for others it is difficult.

Our respondents furthermore described two main strategies for communicating VUSs. One strategy stressed the neutrality of presenting the VUS. This approach was more common among clinicians outside of genetics (oncologists and cardiologists). However, it was also endorsed by a few of the GCs. The second pathway, more common among GCs as well as a few of the physicians, was 'to paint the VUS' (as they called it) by giving the unknown variant a known meaning, described in the following section.

Those HCPs who 'neutralized' the VUS usually described how they deal with VUSs with little detail and little interpretation. Anika, an oncologist, typically described this strategy in the following manner:

I tell the patient: there are here changes A, B, and C. A is defined as pathogenic and B and C are defined as VUSs. I explain what a VUS is, that it's a variant of uncertain clinical meaning, maybe because there is no data about it or maybe because it actually has no meaning.

The oncologist added that she always recommends the patient to have a more detailed consultation with a GC about the VUS if they want to hear more about it.

### 3.4 Down‐ or upgrading the VUS

This section refers to communication after the test. 'Painting the VUS' with the relevant (perceived as closest) meaningful category was a common pathway described by our respondents, especially genetics health professionals. Avi, a medical oncologist, described how in some cases he would ‘downgrade’ a VUS when it is unsuspicious, or ‘upgrade’ it in case of suspicion. Warner, a GC, described his communication strategy in the following manner:

If I see according to prediction models that it looks more benign, I calm the patient down. I tell them that the finding was not identified as prevalent among patients. If I think that a VUS is suspicious, I will say that too.

Many respondents described how they add their assessment of the VUS to the conversation with the patient, upgrading or downgrading the VUS according to their perception. Amy, a GC, typically described this tendency to add meaning when meaning is unknown:

I will give the patient my assessment if it's something that looks more or less suspicious to me. Some VUSs look extremely silly, while other smell very bad. In rare cases, I will say, 'listen we can dig deeper into this, it's a genetic change with potential…'

Importantly, upgrading or downgrading the VUS was described by HCPs as the result of continuing clinical deliberations that considered the relevance of the gene where the VUS was found to the family medical history as well as the ethnic origin and population frequency. Sometimes, depending on the symptomatology, the type of variant, family history, etc., HCPs viewed the VUS as suspicious and thus researched it through segregation studies. Often, following segregation analyses suggesting high frequencies of a VUS among symptomatic family members or other patients, HCPs would paint the VUS as likely pathogenic.

Anika, an oncologist, typically explained how this process is communicated to patients:

I called a patient [after the consultation], and she got scared, so I told her 'no, it's all good’. I told her there was a VUS in the ATM gene and, that VUS was upgraded at the board meeting because the geneticists thought it could be pathogenic. I asked the patient, who was supposed to come to the clinic in three months anyway, that maybe she can come earlier. When she came, I told her that even though it's a VUS, we want to be proactive, leave no stone unturned, and therefore we recommend following up on it like it's not a VUS but a mutation.

HCPs also described how they adapted their VUS communication strategy to the patient's phenotype. Cancer patients, for example, were described by these HCPs as largely indifferent concerning VUSs. Ali, an oncologist, described the influence of the severity of the patient's medical condition on the communicative pathway chosen regarding VUS in the following words:

Some patients see us while they're dealing with active cancer. They are less interested in uncertain genetic changes. If we tell them they are carriers of some VUS, they might want to tell their family members. But at the end of the day, these are patients undergoing chemotherapy, in an acute condition, and they rightfully leave the VUS for later. How much can a person handle? So, often they would be indifferent, and we say 'okay this is something we will handle later on’.

### 3.5 Following up

In post‐test counseling sessions, HCPs faced the challenge of setting up future check‐ins. While some HCPs said they invite the patient to 'come back in a year to see what's going on’, all agreed that initiating effective recontact systematically is impossible given the lack of guidelines, ambiguity about the legal and regulatory requirements, and beyond that, the absence of a computerized database or an information infrastructure where HCPs can be updated about VUSs reclassification. Many HCPs explained that in their communication with patients, they might keep up appearances by telling patients that they are going to check occasionally and that 'these are things that we're looking for, these are the things that we want to learn in the future’. However, they also reflected candidly about the difficulty and contingency of being on top of the reclassification updates. As Frank, a GC, typically explained:

Every clinician can tell their patient, see me again in one year. If a patient is irresponsible and doesn't come back, it's on them. Our responsibility is to explain why it is important. I would not feel bad with myself if I did that and the patient did not follow up.

While following up on reclassification that may lead to recontact was a thorny issue for all the respondents, they usually did not communicate their concerns to the patient. Nevertheless, it remained in the background as a source for potential errors and concerns of personal and systemic constraints among the HCPs.

## 4 DISCUSSION

In this study, we identified how HCPs in genomic medicine handle the uncertainty of genetic variants and examined their strategies for managing and communicating such uncertainty. Whereas variant classification in Israel follows the ACMG guidelines (Li et al., 2017; Richards et al., 2015), no guidelines are in place regarding the way VUS are communicated to patients and the long‐term management of reclassification and recontact past patients with new evidence. Focusing on HCPs' views regarding the daily practice of VUS communication and the perceived gaps between what should and can be done, the current investigation extends research and theory on uncertainty management beyond the perspectives of patients who undergo genetic testing as well as beyond the formal guidelines of doctor–patient communication in personalized genomic medicine. Our findings extend and provide new insights into previous studies of VUSs communication. Zhong et al. (2020) recently found that strategies used by genetic counselors to communicate uncertainty regarding genetic testing results included three core themes: being open and honest, adapting to patients’ needs, and focusing on what is known. Another recent study found that HCP's address VUSs with breast cancer patients through 2 main categories: ambiguity and risk management (Scherr et al., 2020). Ambiguity management included, for example, discussing the opportunity to participate in research studies, clinical DNA banking, expanded genetic testing, additional clinical evaluations, and maintaining contact with the provider who ordered the test. Risk management strategies included discussing potential treatments that are based on objective factors but also account for psychosocial factors such as patients’ preference and emotional response to the medical risk management recommendation. The results of the present study show that GCs and other HCPs involved in communication with patients about VUSs engage in more strategic management through preparing the patient for uncertainty, adapting the level of detail, neutralizing or de‐neutralizing the VUS, and discussing the need to check back. Our respondents did not make a clear distinction in addressing ‘ambiguity’ and ‘risk’ but rather combined them in various ways.

The communication pathways we termed as de‐neutralizing the VUS through interpreting it as likely benign or likely pathogenic, that is, downgrading or upgrading the VUS, can be linked to the institutionalized expectation that it is the role of clinicians to solve diagnostic uncertainties (Brashers et al., 2006; Jutel, 2009). Such expectation can account for the leeway taken by HCPs in interpreting VUSs in a manner that extends beyond the lab report. Furthermore, VUSs reinterpretation by HCPs highlights how dynamic knowledge about genetic variants is used to carve out new medical conditions that are shaping people's lives. Navon (2011, 2019) termed this way of moving from genotype via phenotype to people's lives as ‘genomic designation’. This phenomenon had existed together with the development of the profession of genetic counseling, but has recently increased considerably, not just in volume but also in more fundamental ways.

It is no longer the ideal type of the ‘gene‐for’ or ‘chromosomal abnormality for’ that solely dominates genomic health communication. Instead, HCPs and patients doing precision genomic medicine are faced by a host of genetic variations whose clinical manifestations and disease classification require selective elucidation (Millo et al., 2021; Shkedi‐Rafid et al., 2016). Each VUS is a potential starting point for a process whereby genetic variants are leveraged (Navon, 2011, 2019). Previous influential sociological accounts of the genomic view of the self, such as Paul Rabinow’s (1996) ‘biosociality’ and Nikolas Rose’s (2007) ‘molecular gaze’, were based on and explored already‐existing phenotypically based genetic conditions. Our findings show how in specific cases of higher clinical suspicion VUSs can be constructed as embarking on a potential career of genetic causality.

In that manner, when HCPs tended to look at the VUS as a suspect when they initiated the genetic testing based on severe symptomatology and followed‐up on it with segregation analyses, they were doing uncertainty narration (Timmermans et al., 2017). Such communication efforts that attempt to fit genetic models to data on expressed disease characteristics in biological family members are also illustrative of ‘genomic designation’ (Navon, 2019). In some cases, such narration or designation backfires, as in the story told by the GC about the VUS that was found in a patient who died of a heart attack at a young age, and for years served to guide surveillance in unaffected family members, until the clinical team realized that it might have been a mistake. Such a narrative demonstrates the customization of specific variants through a plot that begins with a mystery, develops through clinical discussions and research analyses, and hopefully ends with a resolution of the causal ambiguity (or not, in this case). It should remind us that providing the patient with an action they could take based on the VUS, which may be recommended for reducing ambiguity (Scherr et al., 2020), does not always correspond with a beneficial coping strategy.

Existing theoretical frameworks on uncertainty communication stress the goal of normalizing uncertainty through mechanisms such as cognitive reappraisal, information seeking, and acceptance (Brashers, 2001; Mishel, 1990). Rather than appraising uncertainty as a threat to their expertise, HCPs engaged in information seeking to make sense of it. In addition to substantiating work on uncertainty management, the current study's findings shed light on the HCPs' concrete strategies for communicating uncertainty. The HCP's strategy of adapting the level of detail to the patient corroborated other adaptation strategies for assessing the individual's informational preferences and capacity for understanding uncertainty (Bansback et al., 2016), including the use of subjective and qualitative descriptions that help to place medical risks in persons' life (Simpkin & Armstrong, 2019). The HCP's strategy of de‐neutralizing the VUS illustrates seeing uncertainty as an opportunity instead of merely a lack of knowledge (Hillen et al., 2017). The HCPs' strategy of preparing the patient for uncertainty in pre‐test counseling is an example of earning patient trust through the open acknowledgment of diagnostic uncertainty (Evans et al., 2009). The pre‐test practice of familiarizing patients with different types of test results, including VUSs, empowers patients to receive uncertain test results that might otherwise be unexpected and confusing. Of note, we found that while consent forms do not offer opting out from receiving VUSs in the oncological and cardiovascular clinical setting, some of our respondents, including GCs, agreed that if the patient expresses a choice not to be told about VUSs, in most cases they will respect that wish, although it would make them uncomfortable. This is an interesting finding that should alert us that the consent form does not necessarily corresponds to the practice. More research is needed to characterize this gap and how it may be addressed.

Finally, our findings suggest that HCPs and not just patients need to cope with uncertainty constantly and deliberately. Indeed, it seems that HCPs have to deal not just with the uncertainty of VUSs but also with another level of uncertainty, related to regulatory or systemic issues regarding, for example, screening policies based on a VUS and the 'joint responsibility' for recontact following reclassification (Levin Fridman et al., 2022). Shifting away from the relatively unilateral process advocated by current frameworks depicting how HCPs' communication of uncertainty affects patients, future research should acknowledge the bilateral negotiation of uncertainty in the interactions between HCPs and patients who collaborate to define the causal ambiguities and boundaries of uncertainty, enacting a care relationship (Lou et al., 2020; Stivers & Timmermans, 2016).

### 4.1 Implications for genetic counseling

With an increasing focus on personalized health care, and advances in genomics and new disease biomarkers, communicating the limitations and errors that come from applying DNA sequencing is an inevitable part of clinician–patient's interactions. This study provides a glimpse into an imminent future where personalized medicine will be inevitably linked to *personalized* communication of diagnostic uncertainties (Eyal et al., 2019), empowering patients and engaging them in decision‐making (Cragun & Zierhut, 2018). Whereas communicating uncertainty to patients has traditionally been constructed in medical training as a sign of weakness/ignorance (Simpkin & Armstrong, 2019), our participants expressed no such views. The more experience HCPs gained in communicating uncertain results, the more likely they were to feel comfortable in communicating them to patients rather than being intimidated by them. Proper pre‐test preparation of patients for the likely possibility of a VUS result will assist patients in adapting to test results and will simplify the post‐test consultation. The major take‐home message to most patients after a VUS result is that classification may change in the future; hence, periodical recontact is recommended. Ideally, automated tools enabling periodical reinterpretation of VUS should be developed and incorporated into clinical care. Until such time arrives, patients are advised to be more involved, for example, via patient registries such as GenomeConnect (Savatt et al., 2021).

### 4.2 Limitations

There are some limitations to this study. Although the participants’ specialties contributed diversity to the sample, the sample size was limited by pragmatic considerations. Other HCPs in other medical centers may express different views. Moreover, findings may be limited to the context of the Israeli health system which is socialized and universal and has a long‐established genetics services as part of public health. Utilizing the present study's qualitative findings, future research should test the effectiveness of HCP's strategies of managing and communicating uncertainty by examining patients’ uncertainty appraisal and perceptions. While our study focused on uncertain genomic findings in the context of cancer and cardio care, future research could also examine the communication of uncertain genomic findings in other settings exploring the possible effect of medical specialty and type of testing on the ways HCPs manage and communicate uncertainty. Despite its limitations, the present study has identified HCPs' strategies in managing and communicating genomic uncertainty.

## AUTHOR CONTRIBUTIONS

Alma Levin Fridman involved in data curation, formal analysis, writing—review and editing, and final approval of the version to be published. Aviad Raz involved in conceptualization, methodology, project administration, writing—original draft, writing—review and editing, and final approval of the version to be published. Stefan Timmermans involved in conceptualization, methodology, review and editing, and final approval of the version to be published. Shiri Shkedi‐Rafid involved in conceptualization, supervision, project administration, methodology, review and editing, and final approval of the version to be published.

## 5 COMPLIANCE WITH ETHICAL STANDARDS

### 5.1 Conflict of interest

Alma Levin Fridman, Aviad Raz, Stefan Timmermans, and Shiri Shkedi‐Rafid do not have any conflicts to report.

### 5.2 Human studies and informed consent

This study has been performed in accordance with the Declaration of Helsinki and has been approved by the Ethics Committee of Hadassah Medical Center #0447‐19‐HMO. Informed consent to participate in the study has been obtained from participants.

### 5.3 Animal studies

No non‐human animal studies were carried out by the authors for this article.

### 5.4 Data availability statement

The datasets generated and/or analyzed during the current study are available from the corresponding author on reasonable request.
